# Improving valuation sampling of EQ-5D health states

**DOI:** 10.1186/1477-7525-11-14

**Published:** 2013-01-31

**Authors:** Adrian Bagust

**Affiliations:** 1 Liverpool Reviews and Implementation Group, University of Liverpool, Liverpool, UK

**Keywords:** Health state valuation, EuroQol, EQ-5D

## Abstract

**Background:**

The original valuation exercise which formed the basis of the UK EQ-5D time trade-off social tariff of health states, employed a sampling scheme involving 43 health states. Neither that study, nor other published international valuations studies have used explicit quantifiable criteria to justify the choice of sampled states. New criteria are proposed and methods described to aid researchers in designing improved sampling schemes for future EQ-5D sampling exercises.

**Method:**

Four such criteria are described, and applied to assess the merits of four sampling schemes previously reported, using three large observational databases to quantify relative performance. An alternative sampling design conforming to these criteria is described, which aims to generate improved performance.

**Results:**

Previous published approaches are shown to perform poorly against the measured criteria. The alternative sampling design is demonstrated to provide superior performance on all measures.

**Conclusion:**

Future valuation exercises using sampled health states based on this approach may be expected to offer benefits in terms of greater precision, avoidance of bias in favour of less severe states, and a higher proportion of research observations valued directly rather than dependent on extrapolation modelling.

## Background

This study was initiated following a seminar given to a multi-disciplinary team of health care researchers outlining the history, development and application of the EQ-5D preference-based measure of health-related quality of life. The audience were all familiar with the use of EQ-5D utility scores in health technology assessment and cost-effectiveness analyses. A number of issues were raised concerning the calibration of scores and their reliability as a central element in decision analysis influencing health policy. Particular areas of concern included the reliability of incremental differences in EQ-5D scores, the inclusion of large negative (worse than death) scores in the UK tariff, and the small number of health states directly valued. During discussion there was especial interest in the states selected for valuation, which did not appear to be balanced by symptom severity or to feature those stages most commonly occurring in clinical trials or clinical practice. As a consequence, the basis for sampling health states has been explored further and the findings are reported here.

Health utility measures are routinely employed by reimbursement agencies in several countries (including UK, Canada, Australia and Sweden) to contribute to the economic evaluation of novel health interventions. The development of the EQ-5D instrument in the UK, was commissioned by the Department of Health and involved a large valuation study (based on a time trade-off (TTO) methodology) in order to inform the assignment of a system of social values to the 243 health states defined by responses to the EQ-5D questions [1].

The original valuation of EQ-5D states was undertaken by the Measurement and Valuation of Health (MVH) group at the University of York, using data gathered from a sample of the general population in the UK. In reporting results of the exercise, Dolan [2] described the issues considered when selecting the 42 states (43 with the healthy state ‘11111’) for use in deriving TTO weightings as follows:

“In choosing the states both for use in the study itself and for each respondent, the most important consideration was that they should be spread widely over the valuation space so as to include as many combinations of levels across the five dimensions as possible. This was subject to the constraint that the states were likely to be considered plausible by respondents.”

However, Dolan [2] did not suggest explicit criteria for assessing the appropriateness and adequacy of any suggested subset of the 243 possible health states for determining weightings, either in relation to the extent of spread across the valuation space, nor for inherent plausibility. Williams [1] provided more detail on four factors taken into account by the MVH researchers:

1) “.states to be more widely spread over the valuation space in terms of mildness or severity (as indicated from earlier valuation data)”

2) “.states to include all plausible combinations of ‘levels’ across each of the 5 dimensions, so as to be able to test for significant interaction effects”

3) “.we wanted to stay as close as possible to the selection of states that had been used by Finnish EuroQol colleagues”

4) “.we wanted to exclude states which seemed prima facie implausible to respondents”

It appears that the dominant issues considered in selecting sample health states were desirable features to assist in modelling valuation data, convenience, and the credibility of scenarios as perceived by valuation respondents drawn from the general population. No mention was made of the potential uses to be made of the resulting EQ-5D utility values, for valuing health gains (or losses) experienced in very different populations; for patients with existing specific health problems, for people in the general population with mainly few adverse symptoms, or for those acutely ill. Sampling states to reflect typical conditions of any of these groups risks distorting the spread of selected states away from those relevant to other groups, increasing the scope for bias in modelled scores. However, without access to extensive data collection from such populations completing the EQ-5D form it would not have been possible to assess whether the MVH approach to selecting health states for valuation was optimal.

Published valuation exercises in Spain [3] and US [4] adopted the same EQ-5D states as used in the UK MVH study, apparently to allow direct comparison with the UK tariff values, but without any comment on their suitability. A Danish study described by Wittrup-Jensen [5] used a modified version of the MVH scheme, omitting four of the original 43 MVH states and adding eight new states, though without providing a clear justification. In Germany [6] a slightly reduced set of MVH health states was employed excluding seven of the original 43 states. Valuation exercises in Japan [7] and the Netherlands [8] adopted a different ‘minimal’ approach using the more limited subset of 17 states (18 including the healthy state ‘11111’) suggested by Macran and Kind [9] as the minimum required to allow full valuation with respondents valuing all states in a single session. It is important to note that both Lamers [8] and Macran & Kind [9] consider only how to reduce the original 42 MVH states (plus ‘11111’) to a smaller subset to make collection of valuation data less onerous and more statistically efficient. It does not address the wider question of whether the 42 states are all appropriate or optimal against other criteria. Table 1 shows a full comparison of the health states used in these studies.

**Table 1:** EQ-5D health states selected for published time trade-off valuations

**EQ-5D health state**	**Health states present in each valuation study**			
	**UK / Spain / US**	**Denmark**	**Germany**	**Japan / Netherlands**
11111	Yes	Yes	Yes	Yes
11112	Yes	Yes	Yes	Yes
11113	Yes	Yes	Yes	Yes
11121	Yes	Yes	Yes	Yes
11122	Yes	Yes	Yes	No
11131	Yes	Yes	Yes	Yes
11133	Yes	Yes	Yes	Yes
11211	Yes	Yes	Yes	Yes
11212	No	Yes	No	No
11221	No	Yes	No	No
11312	Yes	Yes	Yes	Yes
12111	Yes	Yes	Yes	Yes
12121	Yes	No	Yes	No
12211	Yes	Yes	Yes	No
12222	Yes	Yes	Yes	No
12223	Yes	Yes	Yes	No
13212	Yes	Yes	Yes	No
13311	Yes	Yes	No	Yes
13332	Yes	No	No	No
21111	Yes	Yes	Yes	Yes
21121	No	Yes	No	No
21133	Yes	Yes	Yes	No
21221	No	Yes	No	No
21222	Yes	Yes	Yes	No
21232	Yes	Yes	Yes	No
21312	Yes	Yes	No	No
21322	No	Yes	No	No
21323	Yes	Yes	Yes	No
22112	Yes	Yes	Yes	No
22121	Yes	Yes	No	No
22122	Yes	Yes	Yes	No
22222	Yes	Yes	Yes	Yes
22233	Yes	Yes	No	No
22322	No	Yes	No	No
22323	Yes	Yes	Yes	No
22331	Yes	Yes	Yes	No
22333	No	Yes	No	No
23232	Yes	Yes	Yes	Yes
23313	Yes	Yes	No	No
23321	Yes	Yes	Yes	No
32211	Yes	Yes	Yes	Yes
32223	Yes	Yes	Yes	Yes
32232	Yes	Yes	Yes	No
32313	Yes	Yes	No	Yes
32331	Yes	Yes	Yes	No
33212	Yes	No	Yes	No
33232	Yes	Yes	Yes	No
33321	Yes	Yes	Yes	No
33322	No	Yes	No	No
33323	Yes	No	Yes	Yes
33333	Yes	Yes	Yes	Yes
Other states	No	No	No	No

EQ-5D dimensions (left to right): Mobility, Self-care, Usual activities, Pain/discomfort, Anxiety/depression.

Response codes: 1=‘No problems’, 2=‘Some problems’, 3=‘Extreme problems’.

It appears that, in applying the MVH EuroQol paradigm in non-UK settings for TTO valuations, the original selection of 43 states from the available 243 has been modified or reduced for reasons of practicality and local convenience, but has not been reassessed against clearly defined objectives and measurable criteria. The aim of this study is to define such objectives and criteria in the light of large sets of EQ-5D data collected from residents of the UK and four other European countries, and to illustrate how they may be applied to identify more relevant and efficient sampling schemes. It is hoped that the approach outlined and the methods illustrated may prove useful to practitioners seeking to revalue an existing implementation of EQ-5D, or to create a new EQ-5D valuation set.

### Proposed criteria

Four new criteria are proposed which are measurable using empirical data from surveys of individual EQ-5D responses, and provide a basis for comparison of alternative health state sampling schemes. These are based on a reinterpretation of the criteria originally employed by Williams and colleagues [1] other than the MVH desire to align their work closely to that previously done in Finland. The final new criterion develops the implicit assumption in the motivation for the MVH project to be able to detect value differences at the level of definition of the 3-point rating scale of each of the 5 EQ-5D dimensions.

#### Criterion 1: health state plausibility

For a health state to be considered plausible, members of the general public involved in a valuation exercise must be able to conceive of some circumstances which might give rise to a particular pattern of responses to the five EQ-5D questions. This is not a very well defined notion since it depends on the imaginative abilities of individuals, mostly in normal health, who have never personally experienced moderate or severe health problems. Nonetheless, the notion of plausible or implausible states is still meaningful. Answers given to the five EQ-5D questions by someone suffering a specific health state are unlikely to be independent, since correlations between all five dimensions are feasible, and for some pairs are highly probable. This implies that some patterns of response will be very common, and others very rare to the point of being effectively censored. It would be possible to specify a set of health states for a valuation survey which related only to theoretical response patterns which are never seen in real life because of such censoring. It is difficult to see how any credibility could be attached to modelled utility values derived from such an exercise. However, there is currently no *a priori* basis specified for attaching an explicit ‘plausibility’ rating to heath states. Examination of the EQ-5D health states valued by the MVH group, suggests that the means of implementing the plausibility test which was applied involved excluding states which exhibited one of three prohibited pairwise responses (from a total of 90 possible response pairs):

1) Mobility = 3 with Self-care = 1

2) Mobility = 3 with Usual activities = 1

3) Self-care = 3 with Usual activities = 1

This choice appears somewhat arbitrary, and is unsupported by any objective evidence. It is proposed here that the most practical proxy measure for distinguishing plausible from ‘probably implausible’ states is through the analysis of large empirical data sets from survey responses obtained from different patient or resident populations. Where valuation study respondents fail to identify with presented scenarios based on health states that do occur in these populations, the problem should be viewed as one of adequately describing these health states rather than that these states are inherently implausible.

#### Criterion 2: health state relevance

A related question is the relevance of health states chosen for direct valuation to the circumstances and populations in which the derived value estimates (across all health states) are likely to be employed to inform decision-making. Since EQ-5D is essentially a method of capturing aspects of ill-health (rather than health), and is intended for use in assessing the situation of people requiring or receiving remedial health care, preference in the choice of states for direct valuation should be accorded to those states most frequently reported by such people. This should ensure that the proportion of individuals completing the EQ-5D questionnaire in any research study for whom direct valuation has not been employed would be minimised, limiting the additional uncertainty associated with extrapolation modelling.

#### Criterion 3: coverage of health state severity range

In reporting the original MVH study, both Williams [1] and Dolan [2] require that health states “.should be spread widely over the valuation space.” However, translating this statement into a useful index is not straightforward. A count could be made of the number of possible responses for each pair (or triplet) of EQ-5D questions represented at least once in the chosen set of health states. However this is not a very discriminating test, since it only weakly relates to the severity of the overall health state, which is the basis on which the respondent assesses relative utility. If we wish to consider overall health state severity, a single index is required combining information from all five EQ-5D questions, but without prejudging respondents’ preferences. A relatively simple alternative is to use for a particular health state the total number of simple increments (i.e. from 1 to 2 or from 2 to 3) required across all five dimensions to move from the healthy state, giving a severity index ranging from 0 (state ‘11111’) to 10 (state ‘33333’). Thus in designing a sampling scheme, included health states should be evenly spread across all these eleven severity levels.

#### Criterion 4: direct valuation of simple severity increments

Ideally, the ranking and valuation of health states should be based on maximising the opportunities for pairs of adjacent states exhibiting only one response-level difference to be valued by the same respondent. This would yield direct measurement of preferences at the lowest level of differentiation afforded by the EQ-5D instrument. In practice this could only be achieved if every valuer were asked to consider most of the 243 states, which is clearly unrealistic. However, any selected valuation subset of health states can be assessed in terms of the number of potential single difference comparisons which are available for presentation to valuers, so that the valuation scheme is rated more highly where more such pairs of states exist in the valuation space.

## Methods

### Assessment metrics

The extent to which any health state valuation sampling scheme meets the requirement that chosen health states should be both plausible and relevant was assessed by calculating the overall coverage of observations recorded in relevant patient reported surveys and registers by the sampled states. To assess plausibility only states exhibiting non-zero frequency were included, but for relevance the proportion of all responses featuring in the selected valuation health states were calculated. As far as possible selected health states should be evenly spread across the intermediate severity levels (severity index 1–9).

The opportunities present within a subset of EQ-5D health states for direct valuation of single increments in a single dimension were represented as the sum of the number of such links associated with each health state in the selection. If links are represented graphically as lines joining pairs of states, a ‘valency’ number was associated with each state as the number of links starting or ending at the state. The total valency of the sampling scheme is then twice the number of pairwise simple links, and represents a measure of the ‘simple-connectedness’ of the valuation sampling scheme.

### Alternative scheme construction

When multiple criteria are used to assess performance of a sampling scheme it is unlikely that a globally optimal scheme can be identified. However, a scheme has been constructed which addresses all stated objectives in order to illustrate the advantages which are achievable over the approaches employed in the currently published EQ-5D valuation studies.

An iterative procedure was followed to identify candidate states: firstly, all 243 states were ordered in descending frequency of occurrence in the HoDAR data set [10]. Then health states were selected sequentially to fill the quota of five states for each severity level (plus ‘11111’ and ‘33333’). The potential interconnectedness of all states to at least one state at the next higher severity level, and at least one at the next lower level was assessed. Where states failed this test, they were substituted by alternative states (from those unselected states with high frequency), until a well connected network was achieved. Finally, the states within each severity level were allocated to five separate connected streams for valuation by subgroups of respondents. In one case a state could not be allocated without breaking a link in one of the five streams, but this was resolved by identifying a substitute state with a non-zero frequency of occurrence.

## Results

### Performance of reported valuation schemes

The performance of the health state selection schemes detailed in Table 1 was assessed according to three standards.

#### Plausibility and relevance

To assess plausibility and relevance, three observational population data sets of EQ-5D responses were analysed: the Health Survey for England 1996 (HSE96) [11] (a general population sample, n=15476), the CODE-2 study (a disease-specific cross-sectional sample across five European countries, n=4254) [12] and HoDAR (a generic UK hospital population sample, n=43144) [10]. For each health state sampling scheme, the proportion of all recorded responses in each data set which would be directly valued was calculated. In addition, the number of directly valued states for which no observed responses occurred was recorded as an indicator of the plausibility of the selected sampling scheme; if no instances of such a response pattern have been observed the pattern may be inherently unrealistic.

In all of the published valuation studies several of the selected health states (12-28%) do not feature at all in the HSE96 population survey [11]. (Table 2) This is to be expected as the more severe health states, which are required for valuing the full range of potential EQ-5D states, will be quite uncommon in the general population. However, all of the valuation studies also featured health states which did not occur in the two patient population surveys. This suggests that some of these health states may relate to very rare or even completely implausible patterns of response. The original MVH sampling scheme was the least successful on this criterion.

**Table 2:** Plausibility and relevance of published sampling schemes using data from three empirical surveys

	**Valuation study**			
	**UK / Spain / US**	**Denmark**	**Germany**	**Japan / Netherlands**
States valued^#^	43	47	36	18
States valued but not present in observational data set /implausible				
HSE96	12 (28%)	10 (23%)	9 (21%)	5 (12%)
CODE-2	9 (21%)	7 (16%)	5 (12%)	4 (9%)
HoDAR	3 (7%)	2 (5%)	1 (2%)	2 (5%)
Coverage of total responses in observational data set /relevance				
HSE96	82%	88%	82%	74%
CODE-2	63%	78%	68%	49%
HoDAR	58%	75%	57%	44%

# including ‘no problems’ state (11111).

HSE96 Health Survey for England 1996 [11].

CODE-2 Cost of Diabetes in Europe: Type-2 [12].

HoDAR Health Outcomes Data Repository [10].

The proportion of population survey responses covered by the sampling schemes used in the UK, the USA, Spain, Denmark and Germany are quite similar (Table 2), though it appears that the modifications introduced in Denmark were successful in improving direct coverage of both the disease-specific and general acute patient populations. The reduced ‘minimal’ set of states used in Japan and the Netherlands shows disappointing performance for both patient populations, with less than 50% of observations covered by direct valuation.

#### Severity range

The distribution of valued health states by severity index is displayed in Table 3. In both absolute and relative terms the pattern of coverage is not systematic, and is generally biased toward the less severe states. The ‘minimal’ scheme (used in Japan and the Netherlands) has two severity levels in the more serious half of the range with no selected health states. It is also noticeable that all schemes include all five states at level 1, but none uses more than one state at level 9 - its conjugate level. This suggests that incremental valuation differences at lower severity levels are likely to carry more weight than those at higher levels in resulting valuation models.

**Table 3:** Severity coverage comparing number of states included with number available for each severity level

		**Health states present in each valuation study**			
**Severity index**	**EQ-5D states**	**UK / Spain / US**	**Denmark**	**Germany**	**Japan / Netherlands**
0	1	1 (100%)	1 (100%)	1 (100%)	1 (100%)
1	5	5 (100%)	5 (100%)	5 (100%)	5 (100%)
2	15	5 (33%)	7 (47%)	5 (33%)	2 (13%)
3	30	3 (10%)	4 (13%)	2 (7%)	1 (3%)
4	45	8 (18%)	8 (18%)	6 (13%)	3 (7%)
5	51	4 (8%)	5 (10%)	4 (8%)	1 (2%)
6	45	4 (9%)	4 (9%)	4 (9%)	0 (0%)
7	30	10 (33%)	9 (30%)	6 (20%)	3 (10%)
8	15	1 (7%)	3 (20%)	1 (7%)	0 (0%)
9	5	1 (20%)	0 (0%)	1 (20%)	1 (20%)
10	1	1 (100%)	1 (100%)	1 (100%)	1 (100%)
All	243	43 (18%)	47 (19%)	36 (15%)	18 (7%)

#### Simple increments

**Figure 1:**
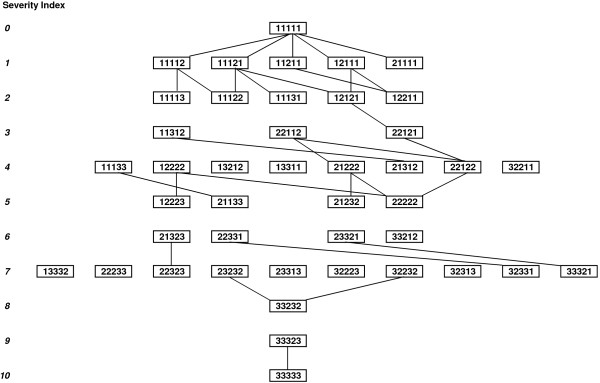
**States in UK, Spanish and US valuations, showing simple direct comparisons available between states. **Solid lines between states are simple comparisons where only one dimension is different by a single rating step. Whether these states are valued by the same valuers depends on the design of the selection scheme chosen to construct manageable valuations subsets. EQ-5D dimensions (left to right): Mobility, Self-care, Usual activities, Pain/discomfort, Anxiety/depression. Response codes: 1=‘No problems’, 2=‘Some problems’, 3=‘Extreme problems’.

The original MVH sampling scheme (as used for valuation in the UK, the US and Spain) is illustrated as a connected network in Figure 1. This reveals that some states are multiply connected, whilst others have no simple links to other states. Since the total number of states is too great for any individual to value, these links will only occasionally result in valuation by the same valuer depending on the way that manageable subsets are selected. It is more concerning that some groups of states with the same severity index are completely disconnected from other severity levels, ensuring that simple direct comparisons are impossible for such health states.

Table 4 summarises the valency totals for the four reported sampling schemes, analysed by severity index. In all cases the preponderance of selected health states with lower severity is mirrored in the distribution of each sampling scheme’s total valency. Although the adapted scheme used in Germany shows similar connectedness to the original MVH sampling scheme, the Danish approach is more successful with 33% more simple links (36% more links per state). By contrast, the ‘minimal’ valuation sampling scheme (Japan and the Netherlands) exhibits very few links between health states, most of those having the lowest severity indices. However, it must be borne in mind that the ‘minimal’ scheme is designed for valuation of all states by every participant, so all links are guaranteed to be present in the collected data. For the other schemes, the number of simple links which are realised in practice is dependent on the design efficiency of random allocation of states to valuers or to separate subsets of health states for valuation by panels of participants, and some loss of valency coverage is to be expected.

**Table 4:** Total and average valency per health state for reported sampling schemes analysed by severity index

		**Valency (valency/state) of health states present in each valuation study**			
**Severity index**	**EQ-5D states**	**UK / Spain / US**	**Denmark**	**Germany**	**Japan / Netherlands**
0	1	5 (5.00)	5 (5.00)	5 (5.00)	5 (5.00)
1	5	13 (2.60)	17 (3.40)	13 (2.60)	7 (1.40)
2	15	9 (1.80)	16 (2.29)	8 (1.60)	1 (1.00)
3	30	5 (1.67)	8 (2.00)	1 (0.50)	0 (0.00)
4	45	10 (1.25)	12 (1.50)	7 (1.17)	0 (0.00)
5	51	6 (1.50)	11 (2.20)	6 (1.50)	0 (0.00)
6	45	3 (0.75)	7 (1.75)	3 (0.75)	-
7	30	5 (0.50)	9 (1.00)	5 (0.83)	0 (0.00)
8	15	2 (1.00)	5 (1.67)	2 (2.00)	-
9	5	1 (1.00)	-	1 (1.00)	1 (1.00)
10	1	1 (1.00)	-	1 (1.00)	1 (1.00)
All	243	60 (1.40)	90 (1.91)	52 (1.44)	16 (0.89)

Valency: the number of simple direct links between states.

### Alternative scheme performance

**Figure 2:**
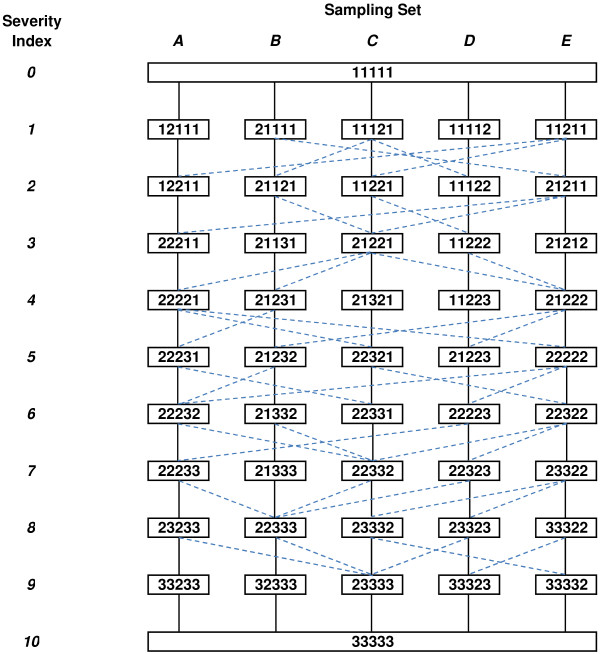
**Alternative valuation scheme showing five panels, and simple direct comparisons between states. **Solid lines between two states are simple comparisons where only one dimension is different by a single rating step, and both states are rated by all members of a rating panel. Dashed lines between two states are simple comparisons where only one dimension is different by a single rating step, and states are valued separately by members of different rating panels. EQ-5D dimensions (left to right): Mobility, Self-care, Usual activities, Pain/discomfort, Anxiety/depression Response codes: 1=‘No problems’, 2=‘Some problems’, 3=‘Extreme problems’.

The illustrative alternative sampling scheme is displayed in Figure 2, with a total of 47 health states arranged as five separate sampling groups each of 11 health states to which death should be added. In each sampling group, the 11 states cover the full range of severity, and are all simply connected to states of greater and lesser severity.

The performance of the alternative sampling scheme is reported in Table 5, and may be compared with the performance of the published schemes (Tables 2 and 3). All valued health states are present in all three observational data sets. All severity levels are equally represented in the selected health states, and the coverage (proportion of observed responses included) for all three data sets is markedly superior to that achievable with the published schemes, both overall and across different severity levels.

**Table 5:** Performance characteristics of illustrated alternative health state sampling scheme

**Severity index**	**States**		**Valency**		**Coverage**		
	**EQ-5D**	**Alternative scheme**	**Total**	**Per state**	**HSE96**	**CODE-2**	**HoDAR**
0	1	1 (100%)	5	5.0	100%	100%	100%
1	5	5 (100%)	15	3.0	100%	100%	100%
2	15	5 (33%)	19	3.8	89%	88%	91%
3	30	5 (17%)	18	3.6	74%	56%	85%
4	45	5 (11%)	19	3.8	84%	76%	91%
5	51	5 (10%)	20	4.0	78%	78%	86%
6	45	5 (11%)	20	4.0	78%	76%	89%
7	30	5 (17%)	20	4.0	89%	67%	93%
8	15	5 (33%)	20	4.0	89%	90%	95%
9	5	5 (100%)	15	3.0	100%	100%	100%
10	1	1 (100%)	5	5.0	-	100%	100%
All	243	47 (19%)	176	3.74	95.3%	85.9%	93.0%

HSE96 Health Survey for England 1996 [11].

CODE-2 Cost of Diabetes in Europe: Type-2 [12].

HoDAR Health Outcomes Data Repository [10].

## Discussion

Since the original MVH valuation study was published by Dolan [2] in 1997, a great deal of research activity has taken place aimed at extending the global reach of the EQ-5D instrument by local implementation, validation and valuation of the EQ-5D instrument. At the same time various different approaches have been developed to improve the methods and statistical modelling required to estimate valuations across the whole EQ-5D state space. However, little attention has been paid to the design of the valuation study providing the raw data from which health state values are derived. This is unfortunate since poorly specified sampling schemes have the potential to conceal implicit inconsistencies in the collected data leading to implausible or imprecise valuations. Of particular concern is the general over-representation of low severity health states, which has the potential to misrepresent the impact of serious conditions with effects on multiple health dimensions.

This study has set out to define criteria for gauging the suitability of a study design for constructing a credible health state valuation sampling set, and to demonstrate how these may be applied to compare the relative performance of different sampling schemes. Since the application of these criteria relies on the availability of a large volume of survey or registry data, it can only be applied after the EQ-5D instrument has become widely used, suggesting that a single valuation study should not be considered the ‘last word’ to define health state values indefinitely, but should be revisited periodically as more empirical data accumulate.

Based on this approach an alternative sample design has been developed to illustrate the gains which are achievable. The resulting scheme uses no more health states than the best-performing published study, [5] and requires members of the public to rate only 12 states each in total (including death and perfect health).

An advantage of this approach for users of the valuations obtained is the assurance that the great majority of observations in any health-related study (whether of health care users or the general public) will have been directly valued (rather than inferred by modelling), and that for rarer health states any modelling is founded on data relating to real rather than hypothetical and possibly infeasible states.

For valuation modelling, the full coverage of all severity levels and simple linkage of all health states valued by each respondent should ensure that inconsistency is reduced and more easily identified. It is likely that estimation uncertainty will also be reduced. Moreover, non-linearity of incremental changes across severity levels could be studied directly at the level of the individual respondent, rather than inferred indirectly.

A key feature of this approach is the redefinition of ‘plausibility’ as a criterion based on the use of empirical data. Though ‘plausibility’ is an important concept of obvious relevance to valuation studies, in none of the published studies has a quantifiable definition been proposed which is proof against challenge. The original MVH group had minimal information on the likely distribution of responses in large populations, and therefore adopted a limited pragmatic test, and later studies have not reconsidered this issue at all. The approach taken here is also pragmatic but has the benefit of being informed by a much richer set of evidence drawn from three contrasting sources. Exclusion of any response pattern with zero recorded responses doesn’t guarantee that all these patterns are impossible, but it is a reasonable filter which does ensure that all included patterns of response have some positive evidence of potential plausibility.

Since the criteria presented here rely on a mixture of intrinsic features of health states, and frequency statistics obtained from empirical sources, the alternative sampling scheme described is not necessarily generalisable to all national contexts. Indeed, it is unlikely that a unique ‘best design’ exists within any context, since there is scope for multiple solutions yielding similar performance levels.

## Conclusion

This study has shown that application of explicit measurable criteria can lead to improved designs for sampling EQ-5D health states in a valuation exercise, provided a sufficient volume of real-life patient EQ-5D responses is available as a basis for identifying appropriate health states. Realising the potential benefits of such sampling schemes will need to be tested in practice.

## Competing interests

The author declares that he has no competing interests

## References

[B1] Williams A The measurement and valuation of health: a chronicle. Centre for Health Economics Discussion Paper 136 1995 University of York

[B2] Dolan P Modeling valuations for EuroQol health states Medical Care 1997 35 1095 1108 10.1097/00005650-199711000-00002 9366889

[B3] Badia X Roset M Herdman M Kind P A Comparison of United Kingdom and Spanish General Population Time Trade-off Values for EQ-5D Health States Med Decis Making 2001 21 7 16 10.1177/0272989X0102100102 11206949

[B4] Shaw JW Johnson JA Coons SJ US valuation of the EQ-5D health states. Development and testing of the D1 valuation model Medical Care 2005 43 203 220 10.1097/00005650-200503000-00003 15725977

[B5] Wittrup-Jensen KU Lauridsen JT Gudex C Brooks R Pedersen KM Estimating Danish EQ-5D tariffs using the time trade-off (TTO) and visual analogue scale (VAS) methods Paper presented at the 18th Plenary Meeting of the EuroQol Group 2001 http://www.euroqol.org/uploads/media/Proc01Copen20WittrupJensen.pdf

[B6] Greiner W Claes C Busschbach JJV von der Schulenburg J-M G Validating the EQ-5D with time trade off for the German population Eur J Health Econom 2005 6 124 130 10.1007/s10198-004-0264-z 19787848

[B7] Tsuchiya A Ikeda S Ikegami N Nishimura S Sakai I Estimating an EQ-5D population value set: the case of Japan Health Econ 2002 11 341 353 10.1002/hec.673 12007165

[B8] Lamers LM McDonnell J Stalmeier PFM Krabbe PFM Busschbach JJV The Dutch tariff: results and arguments for an effective design for national EQ-5D valuation studies Health Econ 2006 15 1121 1132 10.1002/hec.1124 16786549

[B9] Macran S Kind P Badia X, Herdman M, Roset M Valuing EQ-5D health states using a modified MVH protocol: preliminary results16th Plenary Meeting of the EuroQol Group, Sitges, 6–9 November 1999, Discussion Papers 2000 Spain: Institut de Salut Publica de Catalunya 205 240

[B10] Currie CJ McEwan P Peters JR Patel TC Dixon S The Routine Collation of Health Outcomes Data from Hospital Treated Subjects in the Health Outcomes Data Repository (HODaR): Descriptive Analysis from the First 20,000 Subjects. Value in Health 2005 8 581 590 10.1111/j.1524-4733.2005.00046.x 16176496

[B11] Joint Health Surveys Unit of Social and Community Planning Research and University College London Health Survey for England, 1996 [computer file] 2001 3 Colchester, Essex: UK Data Archive [distributor] SN: 3886

[B12] Massi-Benedetti M The cost of diabetes type II in Europe. The CODE-2 study Diabetologia 2002 45 S1 S4 10.1007/s00125-002-0860-3 12136404

